# Bibliometric analysis of the application of artificial intelligence techniques in bacteriology: a PRISMA-guided research agenda

**DOI:** 10.3389/fmicb.2025.1641967

**Published:** 2025-09-16

**Authors:** Diana Carolina Velasco Cardona, Sebastián Cardona-Acevedo, Alejandro Valencia-Arias, Orlando Pérez-Delgado, Melissa Pinella Vega

**Affiliations:** ^1^Ciencias de la Salud, Institución Universitaria Colegio Mayor de Antioquia, Medellín, Colombia; ^2^Facultad de Ciencias Económicas y Administrativas, Instituto Tecnológico Metropolitano, Medellín, Colombia; ^3^Escuela de Ingeniería Industrial, Universidad Señor de Sipán, Chiclayo, Peru; ^4^Vicerrectoría de Investigación, Universidad Señor de Sipán, Chiclayo, Peru; ^5^Facultad de Ciencias de la Salud, Universidad Señor de Sipán, Chiclayo, Peru

**Keywords:** artificial intelligence, bacteriology, machine learning, bibliometric analysis, bacterial classification, metagenomics

## Abstract

**Introduction:**

The integration of artificial intelligence (AI) into bacteriology has marked a pivotal advancement by enabling the analysis of large-scale microbiological datasets. Despite growing adoption, significant research gaps persist, hindering the full exploitation of AI’s potential in bacterial research and diagnostics.

**Objective:**

To analyze global scientific production on the application of AI techniques in bacteriology and propose a future research agenda based on bibliometric trends.

**Methods:**

This study conducts a bibliometric analysis of artificial intelligence (AI) applications in bacteriology, explicitly guided by the PRISMA 2020 framework. Unlike traditional reviews, this approach combines PRISMA’s methodological rigor with bibliometric techniques to map scientific production. Metadata were retrieved from Scopus and Web of Science using predefined search strategies. Quantitative indicators, co-occurrence networks, and thematic mapping were applied to examine the field’s temporal evolution and conceptual structure. The findings provide an evidence-based overview of research trends and gaps, supporting the design of a future research agenda on AI integration in bacteriology.

**Results:**

The findings reveal exponential growth in scientific output, especially between 2022 and 2024. Leading authors include Singh and Waegeman, with high-impact journals such as Frontiers in Microbiology and MSystems. The United States and China are the most productive countries. Thematic evolution shows a shift from early topics like microbial spoilage toward advanced applications including bacterial classification and diagnostic modeling. Key conceptual clusters were identified around microbiomes, classification, and bioinformatics. Emerging terms such as “diagnosis,” “metagenomics,” and “transfer learning” indicate future research directions.

**Conclusion:**

AI applications in bacteriology are expanding rapidly yet still rely heavily on traditional machine learning methods. There is a need to incorporate advanced approaches such as deep learning and transformer-based models. The findings support a strategic agenda for promoting interdisciplinary collaboration and technological innovation in bacteriological research.

## 1 Introduction

The incorporation of artificial intelligence (AI) into bacteriology signifies a pivotal advancement in the comprehension and administration of microorganisms. It facilitates the examination of extensive quantities of microbiological data and enables the discernment of patterns that would otherwise remain obscured with conventional techniques. The utilization of machine learning algorithms has demonstrated efficacy in the analysis of the microbiome and the comparison of diverse diseases, thereby indicating that AI has the potential to transform the study and treatment of bacterial infections (Wirbel et al., 2021).

The term “artificial intelligence” refers to the development of computer systems capable of performing tasks that typically require human intelligence, such as learning, reasoning, and problem solving (Graf et al., 2025). Machine learning (ML) is a term used to describe algorithms that learn patterns from data to make predictions or classifications. A more advanced subset, deep learning (DL), employs artificial neural networks with multiple layers to model complex relationships and has demonstrated considerable potential in image recognition, natural language processing, and biomedical data analysis (Syed et al., 2025). Given the extensive nature of AI, the present study focuses specifically on bibliometric trends related to machine learning and deep learning techniques as applied to bacteriology. The present study does not encompass other AI subdomains, such as robotics, expert systems, and computer vision hardware integration. This delineation facilitates a targeted examination of the manner in which data driven AI methodologies are precipitating a transformation within the domain of microbiology, with a particular focus on domains such as bacterial classification, diagnostic prediction, and microbiome analysis.

Furthermore, machine learning techniques have impacted microbial ecology and environmental monitoring, optimizing the interpretation of complex data and improving the ability to detect changes in microbial composition (Ghannam and Techtmann, 2021). Furthermore, the potential of microbiome analysis to predict soil characteristics has been explored, indicating that AI can exert a significant influence on the health of the microbial ecosystem in agricultural contexts (Wilhelm et al., 2022).

In the field of microbial identification and classification, AI has been shown to enhance the precision of metagenomic data interpretation through the utilization of deep learning models (Fiannaca et al., 2018). This technology facilitates the identification of patterns in microbiome studies, which is essential for understanding microbial interactions and their effects on health (Topçuoğlu et al., 2020). Furthermore, it enables the expeditious assessment of antimicrobial susceptibility through vibrational spectral analysis, thereby facilitating the acquisition of real time results, which is of paramount importance in the battle against bacterial resistance (Thrift et al., 2020).

Bacteriology, a pivotal subfield of microbiology, entails the identification, classification, and characterization of bacteria and their roles in health, disease, and the environment (Hardy et al., 2025). It plays a pivotal role in the fields of infectious disease diagnostics, antimicrobial resistance surveillance, and environmental monitoring. Given its global relevance, the incorporation of computational tools such as AI offers promising avenues to improve accuracy and scalability. In order to comprehend the manner in which artificial intelligence is influencing this domain, bibliometrics a set of quantitative methods for analyzing scientific literature provides significant insights into research trends, influential authors, and emerging topics across time.

Recent scholarly works have addressed the integration of artificial intelligence into microbiologyrelated domains, offering valuable but distinct perspectives. For instance, Xu et al. (2022) conducted a bibliometric analysis focusing on AI in biotechnology and applied microbiology, identifying global collaborations and hot topics. However, their approach remained general and did not isolate bacteriology as a specific field of analysis. In a similar vein, Abdelwahab et al. (2024) presented a thematic based narrative review on AI and microbiome research, emphasizing evolutionary and conceptual patterns. However, they did not employ formal bibliometric techniques. In contrast, Zubair (2024) conducted a systematic review and metanalysis on the use of artificial intelligence (AI) in bacterial infection diagnostics, focusing on clinical implications rather than on scientific production patterns. The present study is distinct from these works in that it employs a targeted bibliometric analysis of AI applications in bacteriology, mapping thematic evolution, conceptual clusters, and emerging research directions. This focused approach provides a unique contribution by clarifying how AI has been adopted within the bacteriological research landscape and identifying critical gaps and opportunities.

Artificial intelligence is transforming bacteriology by enhancing the accuracy, speed, and scalability of diagnostic, classification, and predictive processes. In clinical contexts, AI enables rapid identification of bacterial pathogens through spectral and genomic data analysis, facilitating timely treatment decisions and combating antimicrobial resistance (Thrift et al., 2020). In taxonomy, machine learning models have improved species-level classification, especially in complex metagenomic environments, where traditional methods often lack resolution (Fiannaca et al., 2018). Predictive algorithms also support microbial growth modeling, antimicrobial susceptibility testing, and the simulation of microbial ecosystems, expanding the scope of bacteriology into environmental and agricultural applications. These advances reflect a paradigm shift in which AI-driven systems are not merely auxiliary tools, but integral components in microbial research and clinical workflows (Topçuoğlu et al., 2020).

Traditional bacteriological methods rely on culture-based techniques, biochemical assays, and microscopy. These techniques require extended incubation times, manual interpretation, and highly trained personnel. While these approaches are well-established, they often have limitations in terms of sensitivity, throughput, and scalability. In contrast, AI-driven methodologies enable automated data processing, pattern recognition, and predictive modeling based on genomic, proteomic, or spectral inputs. For instance, Thrift et al. (2020) showed that AI can accelerate antimicrobial susceptibility testing via vibrational spectral analysis, providing near real-time results. Similarly, Bruyne et al. (2011) showed that applying machine learning to MALDI-TOF mass spectra improves the accuracy of bacterial identification beyond conventional taxonomy. Topçuoğlu et al. (2020) further illustrated that AI models can classify microbiome data without predefined assumptions, reducing dependency on experts. Overall, AI techniques surpass traditional approaches in terms of speed, precision, and adaptability to large-scale, multidimensional data. However, their implementation requires computational infrastructure and careful validation.

Nevertheless, despite the considerable progress made in the utilization of AI in bacteriology, there remain significant research gaps that impede a comprehensive understanding and broader application of this technology. Despite the exploration of a range of AI algorithms in different microbiological fields, a comprehensive analysis of their efficacy and suitability for classifying and characterizing bacterial strains is still lacking (Coy-Aceves and Corona-Vasquez, 2022). Similarly, recent studies indicate that, although predictive models have been developed to determine the growth status of bacteria, the implementation of these techniques in unseen strains remains an underexplored challenge (Yamamoto et al., 2023). Consequently, the objective of this research is to explore research trends in this regard. To this end, the following research questions are also formulated:

- Which are the years in which there has been the most interest in the use of AI in bacteriology?
- What type of growth has the number of scientific articles on the use of AI in bacteriology shown?
- What are the main research references on the use of AI in bacteriology?
- What is the thematic evolution derived from the scientific production on the use of AI in bacteriology?
- What are the main thematic clusters on the use of AI in bacteriology?
- What are the growing and emerging keywords in the research field on the use of AI in bacteriology?

The article is structured as follows: an introduction, in which the objectives and context of the study are set out; a methodology section, in which the criteria and tools used are described; a results section, in which the data analysis is presented; a discussion section, in which the findings are interpreted in comparison with previous studies; and a conclusion section, in which the main discoveries are summarized and future research is suggested.

## 2 Methodology

This study is exploratory in nature and based on secondary research sources. A bibliometric analysis was employed to examine the literature on the use of artificial intelligence in bacteriology. The analysis was conducted in accordance with the parameters established by the PRISMA-2020 declaration, with the objective of ensuring an adequate review of the selected sources (Page et al., 2021). The present study is of an exploratory nature and is based on secondary research sources. An exploratory study is a research endeavor that aims to generate insights and identify patterns within a topic that has yet to be thoroughly understood. This study serves as a foundational framework for subsequent, more structured research. The utilization of secondary sources entails the examination of data previously collated and disseminated by other researchers. In this particular instance, the focus is on scientific publications that have been indexed in bibliographic databases.

Bibliometric analysis is a methodological approach that utilizes quantitative methods to evaluate scientific publications. This approach is frequently employed to understand the structure, dynamics, and evolution of a research field. By measuring publication outputs, coauthorships, keyword cooccurrence, and citation patterns, bibliometrics allows researchers to identify influential studies, trace thematic developments, and propose future research agendas. In this study, bibliometric techniques are employed to examine the adoption and development of artificial intelligence methods within the field of bacteriology, thereby facilitating a data driven understanding of this interdisciplinary intersection.

### 2.1 Eligibility criteria

The present study’s inclusion criteria were derived from two fundamental aspects. Firstly, relevant terms were sought in the titles and keywords as primary metadata. The relevant terms included combinations related to artificial intelligence (e.g., “Artificial Intelligence,” “Machine Learning,” “Deep Learning,” “Neural Networks,” “Natural Language Processing”) and bacteriology (e.g., “bacteria,” “microbiology”). These terms were selected based on their frequent occurrence in literature related to both domains.

The exclusion process was conducted in three phases. In the initial phase, all erroneous records were eliminated; these refer to entries in the database that contained incorrect or missing metadata, such as author names, publication years, or journal titles. In the subsequent phase, documents lacking full text access were designated for exclusion; however, this step was not implemented due to the exclusive focus of this bibliometric analysis on metadata rather than full texts. The third phase of the review involved the exclusion of documents with incomplete indexing, such as those lacking author affiliations, country data, or keywords. Additionally, conference proceedings and documents deemed to be of limited relevance were excluded.

The latter term refers to publications that, despite containing AI related terminology, did not specifically address applications in bacteriology or bacterial systems (e.g., articles on fungal pathogens or general algorithmic development without microbiological context). The analysis excluded conference proceedings and preprint articles. This decision was made to prioritize sources that have undergone the peer review process, thereby ensuring a minimum standard of scientific quality and academic validation. While conference papers and preprints are important dissemination channels especially in rapidly evolving fields like artificial intelligence their inclusion could introduce publications that have not undergone formal evaluation, potentially compromising the consistency and reliability of the bibliometric analysis.

### 2.2 Source of information

**Figure 1:**
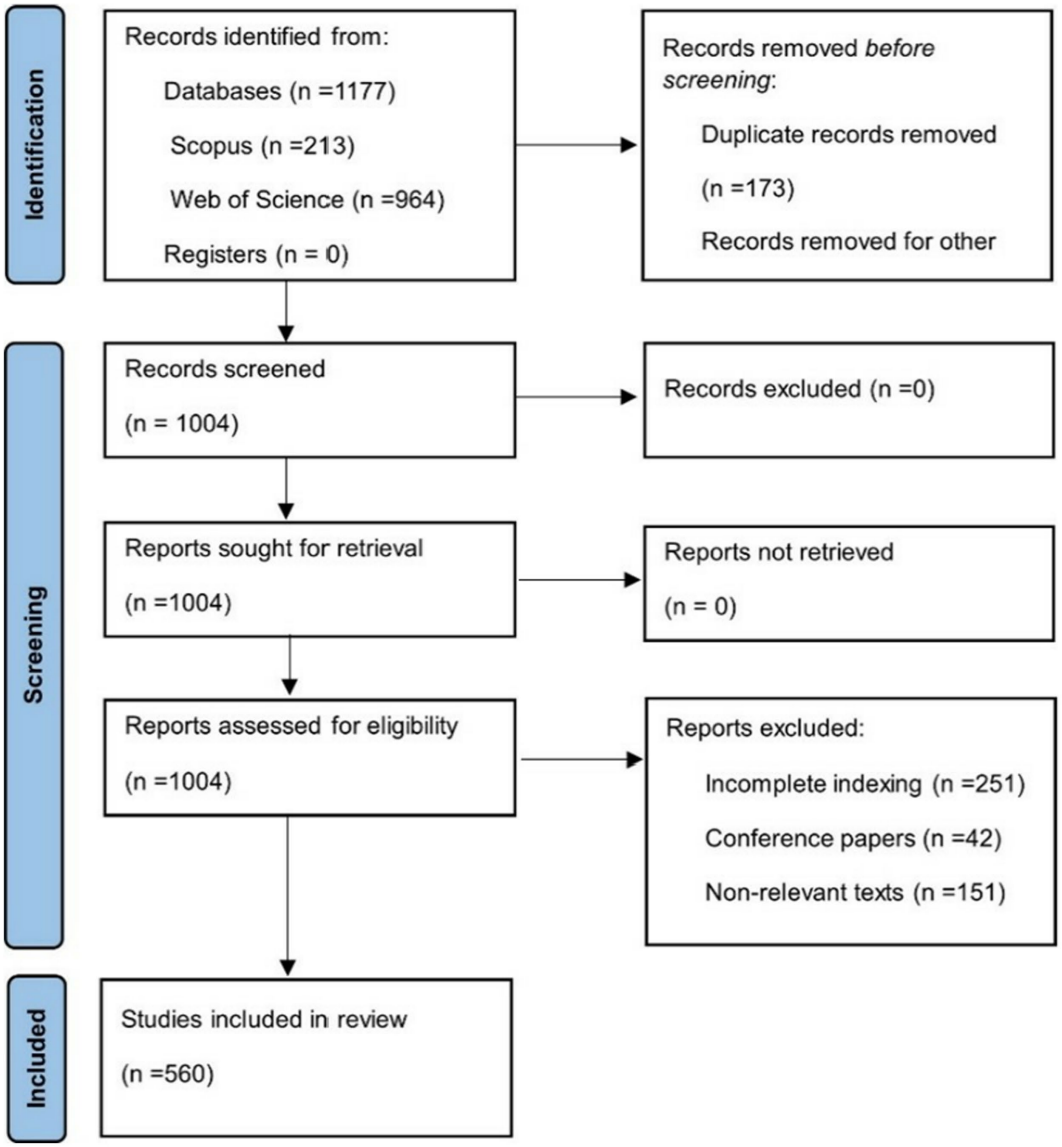
PRISMA flowchart. Own elaboration based on Scopus and Web of Science. PRISMA 2020 flow diagram showing the identification, screening, eligibility, and inclusion process of documents selected for the bibliometric analysis. Records were retrieved from Scopus and Web of Science using predefined search equations. The flowchart illustrates the systematic exclusion phases, including duplicate removal, metadata errors, and incomplete indexing. This ensures transparency in the selection process and methodological rigor in accordance with PRISMA guidelines.

**Figure 2:**
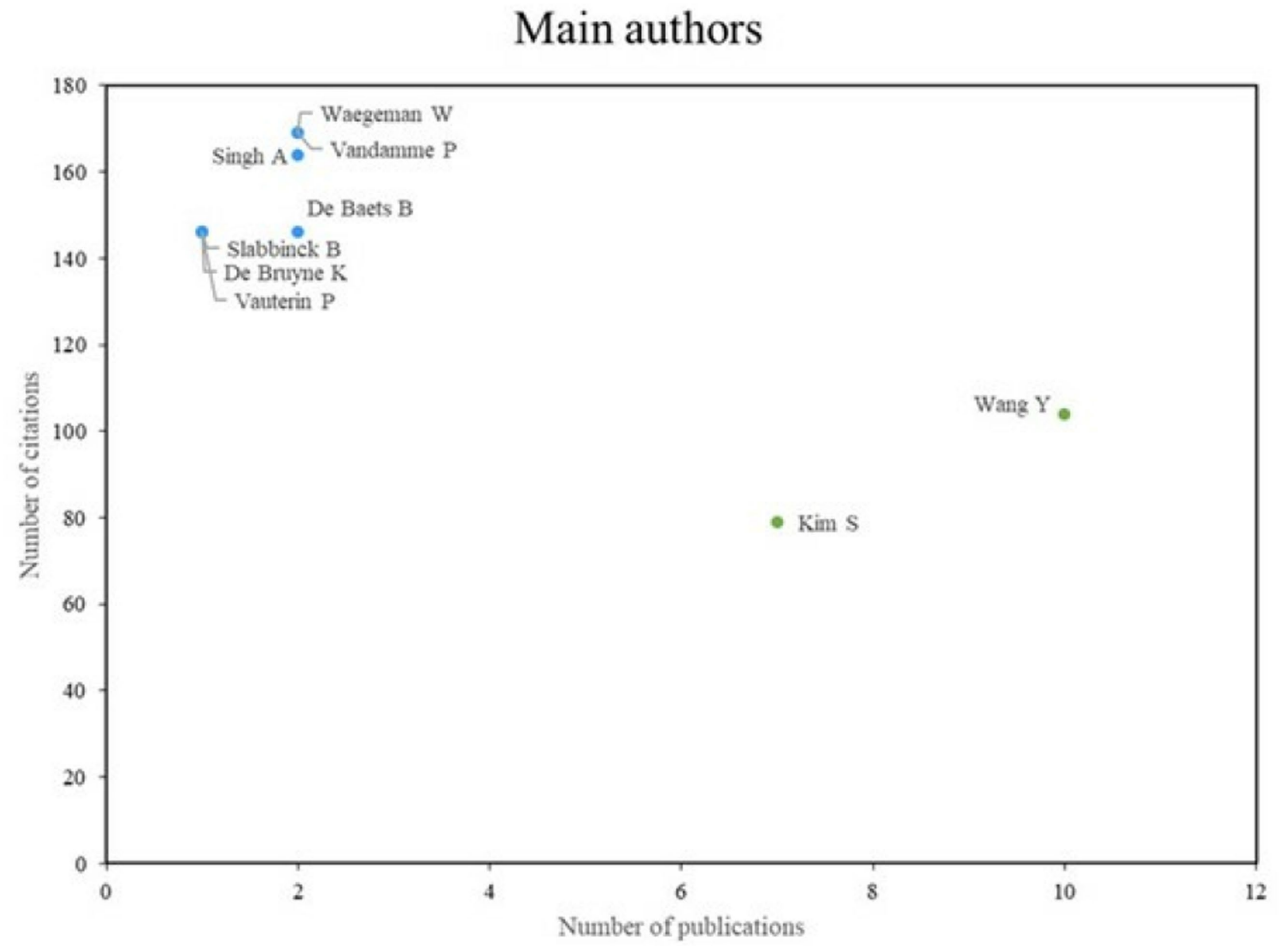
Main authors. Prepared by the authors based on Scopus and Web of Science. Most prolific and influential authors in the field of artificial intelligence applied to bacteriology, based on number of publications and citations. The data were processed from bibliographic metadata obtained through Scopus and Web of Science and analyzed using Excel®. The figure distinguishes between high-impact authors (with fewer but highly cited publications) and highly productive authors (with numerous publications but lower average citations), showing the different forms of scholarly influence in the field.

This bibliometric study exclusively relied on the Scopus and Web of Science (WoS) databases for data retrieval. The decision to restrict the search to these sources was based on their established role as the most comprehensive and reliable platforms for bibliometric research, given their wide citation coverage, rigorous peer-review indexing standards, and provision of high-quality, structured metadata (Gusenbauer, 2024). Using only Scopus and WoS also reduces the risk of inconsistencies and duplicate records that are common in alternative databases, thereby ensuring replicability and comparability of results across studies. The metadata extracted included information on authorship, keywords, countries, journals, and citations. To guarantee methodological transparency, all searches were conducted using predefined strategies documented in accordance with PRISMA 2020 guidelines. The retrieved records were exported in compatible formats and subsequently processed, cleaned, and stored using Microsoft Excel®, which allowed systematic organization for subsequent bibliometric analyses (Figure 1; Figure 2).

**Figure 3:**
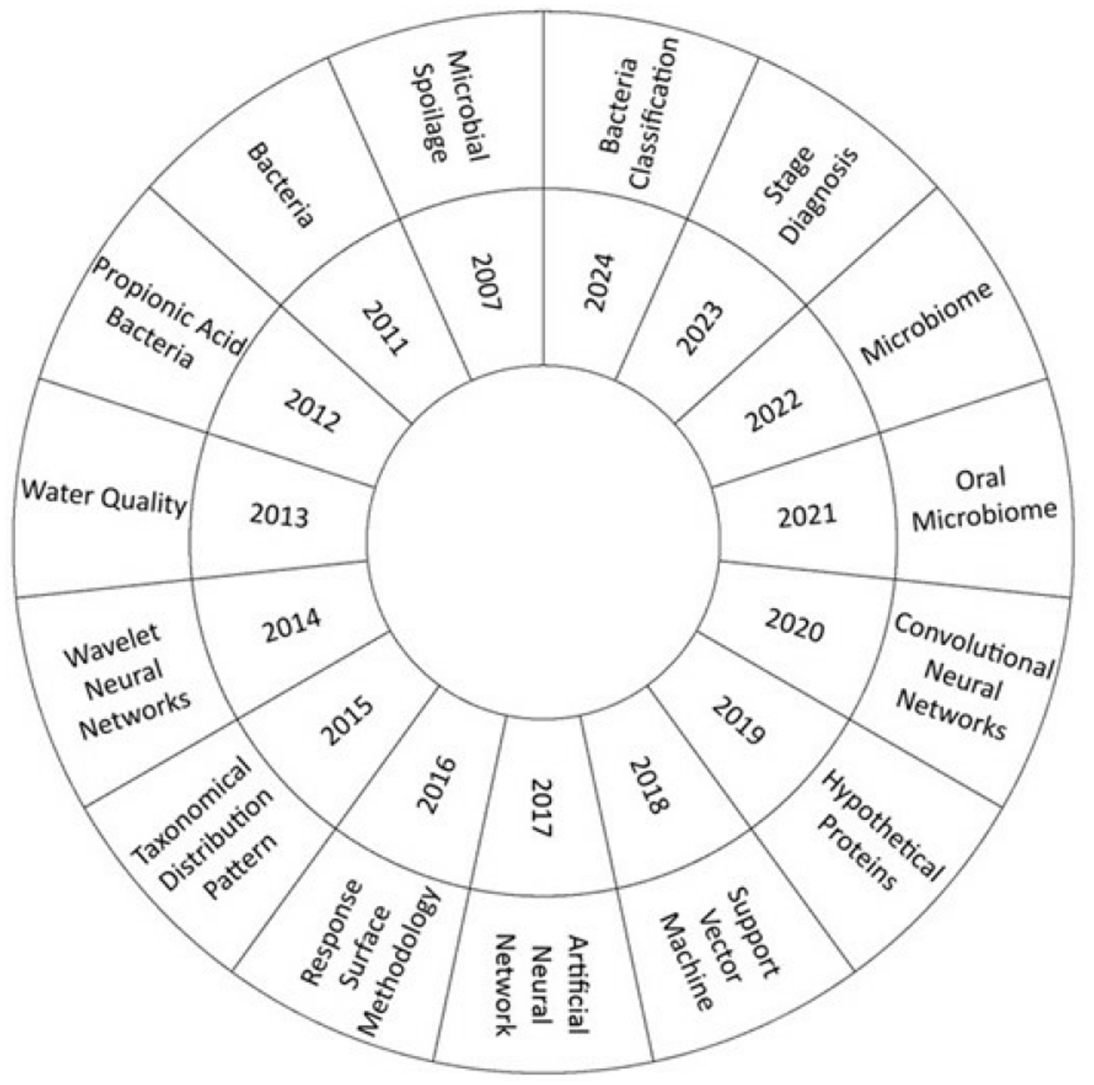
Thematic evolution. Own elaboration based on Scopus and Web of Science. Thematic evolution of keyword usage in publications related to AI in bacteriology from 2007 to 2022. Using VOSviewer, co-occurring terms were mapped over time to identify shifts in research focus. Early terms such as “microbial spoilage” have gradually been replaced by more advanced topics like “bacteria classification” and “microbiome.” This figure illustrates the intellectual progression and growing complexity of the field.

**Figure 4:**
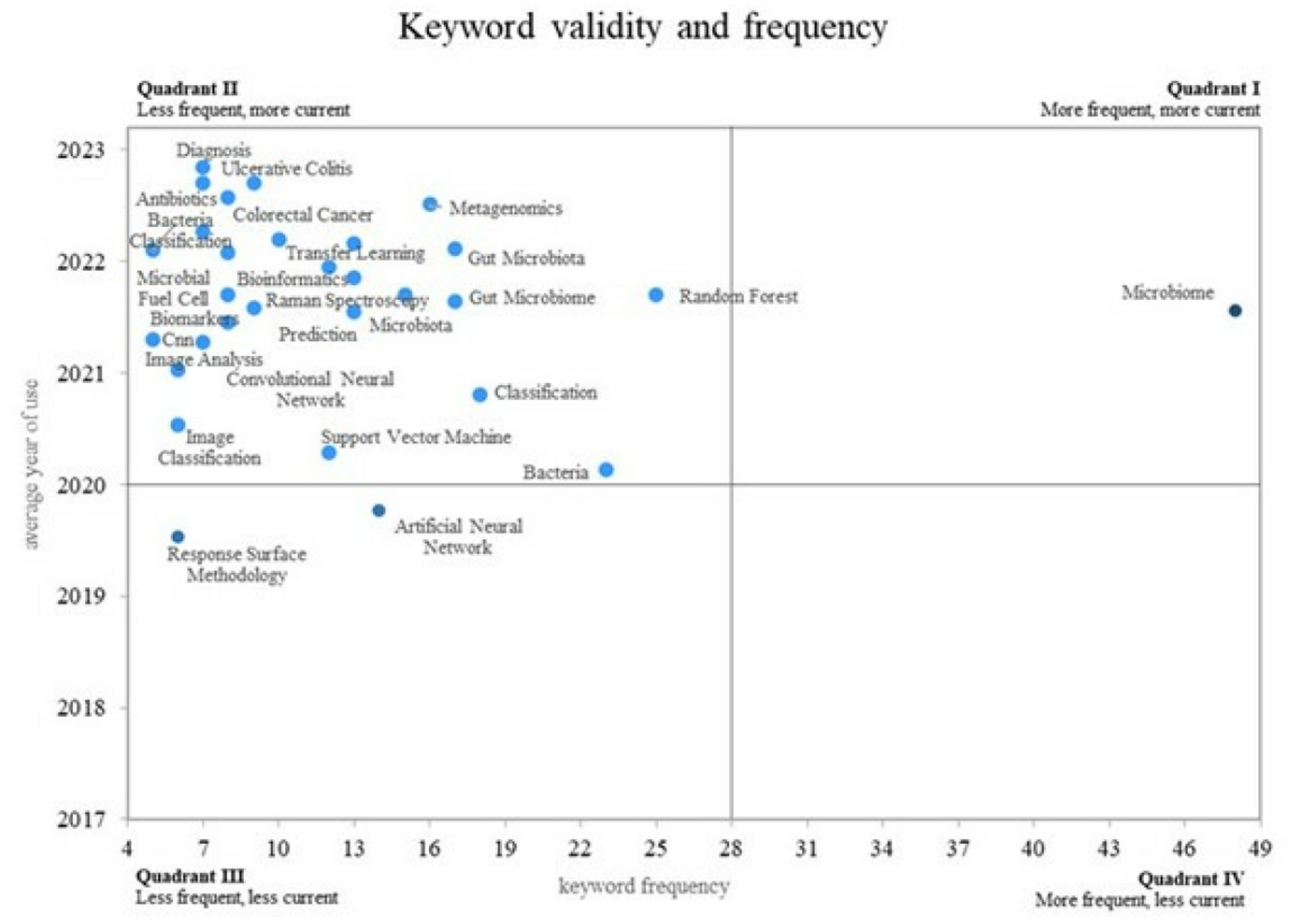
Validity and frequency of keywords. Prepared by the authors based on Scopus and Web of Science. Cartesian diagram mapping keywords according to frequency of use (X-axis) and temporal relevance (Y-axis). The figure classifies terms into four quadrants, with quadrant 1 showing established and growing topics (e.g., “microbiome”), and quadrant 2 highlighting emerging but underexplored concepts (e.g., “metagenomics,” “transfer learning”). This analysis helps identify research trends and gaps for future exploration.

Figures 3, 4 were generated using VOSviewer (version 1.6.x) and Microsoft Excel®, based on metadata retrieved from Scopus and the Web of Science database. Figure 3 shows the results of a co-occurrence analysis of author keywords performed using VOSviewer. A minimum occurrence threshold of five keywords was applied to ensure thematic relevance. The keywords were standardized manually to merge lexical variants (e.g., “AI” and “Artificial Intelligence”). The temporal overlay visualization was selected to reflect the average publication year of each keyword. Figure 4 is a Cartesian plot constructed in Excel® using two variables: frequency of keyword occurrence on the X-axis and average publication year as a proxy for conceptual validity on the Y-axis. Frequency values were obtained directly from the VOSviewer output, and average year values were calculated using weighted means based on article counts per keyword. The quadrant structure was defined based on relative medians to differentiate between established, emerging, and declining concepts.

The visualization formats selected were chosen for their effectiveness in revealing thematic evolution, conceptual clustering, and temporal relevance within a complex, multidisciplinary research field. Figure 3 uses a co-occurrence map with a temporal overlay to show shifts in research focus over time and highlight emerging and declining topics. Figure 4 uses a Cartesian diagram to represent the frequency and recency of keyword usage, clearly differentiating between well-established, emerging, and obsolete concepts. These formats were preferred over purely statistical summaries because they facilitate the intuitive interpretation of trends and support strategic research planning based on thematic positioning.

### 2.3 Search strategy

To search the two selected databases, two bespoke search equations were devised, crafted to align with both the defined inclusion criteria and the search particularities of each database.

For the Scopus database: (TITLE (bacter* OR microbi*) AND TITLE (“Artificial intelligence” OR “Machine Learning” OR “Deep Learning” OR “Neural Networks” OR “Natural Language Processing”)).

For the Web of Science database: (TI = (bacter* OR microbi*) AND TI = (“Artificial intelligence” OR “Machine Learning” OR “Deep Learning” OR “Neural Networks” OR “Natural Language Processing”)).

### 2.4 Data management

The Microsoft Excel® tool was employed for the extraction, storage and processing of information derived from a variety of databases. Furthermore, the open source software VOSviewer was employed for the visualization and analysis of bibliometric networks, in conjunction with Microsoft Excel for the generation of graphs representing various bibliometric indicators. The software was developed by Eck and Waltman (2010), who describe its utility for bibliometric mapping.

### 2.5 Data collection process

The methodology employed for the acquisition of data from the records retrieved from the two selected databases is described below. Microsoft Excel® was utilized as a tool to automate the extraction and organization of bibliographic metadata.

All authors participated in the data validation process through cross-review procedures. In these procedures, each researcher was assigned a specific role, such as data extraction, coding, or consistency verification. Then, the researchers reviewed the outputs of their peers to ensure accuracy. Although the authors were not external to the study, this internal cross-validation helped reduce potential bias and maintain methodological rigor.

Subsequent to the preliminary cross-review phase, an iterative group validation process was executed, wherein the authors collaboratively examined and deliberated on discrepancies in the extracted data. This procedure was continued until full consensus was reached among all researchers regarding the accuracy and consistency of the final dataset. This collaborative approach ensured a high level of reliability in the data used for bibliometric analysis.

### 2.6 Assessment of the risk of bias of the study

As with the methodology employed for data collection, the methods used to assess the risk of bias in the included studies are also specified. The aforementioned process was conducted utilizing the same automated tool, Microsoft Excel®, employed during the data collection phase. All authors participated in the assessment of the risk of bias, working collectively to guarantee the quality and integrity of the results obtained. The utilization of Excel® facilitated the effective systematization of the information, thereby ensuring a rigorous and coherent analysis.

### 2.7 Synthesis methods

The processes employed to ascertain the eligibility of the studies in accordance with the characteristics of the intervention and its comparison with the planned groups are also delineated. These planned groups were defined based on bibliometric performance, allowing a classification of authors, journals, and countries into categories such as (Wirbel et al., 2021): high productivity and high impact (e.g., Frontiers in Microbiology; Graf et al., 2025), high impact but low productivity (e.g., Computational and Structural Biotechnology Journal), and (Syed et al., 2025) high productivity but moderate impact (e.g., MSystems). This classification facilitated a more nuanced interpretation of contribution types.

Methods were implemented to prepare data for presentation, including the handling of missing summary statistics and data conversions, thus ensuring the integrity of the information. For example, 14 articles lacked author country affiliations and 27 records were missing keyword descriptors these were excluded in the third phase of filtering. In terms of data conversion, inconsistencies in publication years (e.g., “0000” or blank entries) were manually corrected through DOI lookups. Keywords were also standardized, merging variants such as “AI” and “Artificial Intelligence” to improve the accuracy of the co-occurrence analysis.

Furthermore, bibliometric indicators of quantity, quality, and structure were applied automatically using Microsoft Excel® to all documents that passed the three exclusion phases, guaranteeing a systematic and rigorous analysis (Durieux and Gevenois, 2010). Quantity indicators included metrics such as the number of publications per year, author, journal, and country (e.g., 126 documents published in 2024). Quality indicators were based on citation counts, highlighting impactful contributors like Singh A., who received over 450 citations. Structural indicators focused on network relationships (e.g., co-authorships and keyword clusters), and were visualized using VOSviewer to uncover conceptual and collaborative patterns in the field.

The preliminary identification phase was conducted through a systematic search strategy across the selected information sources, followed by the removal of duplicate records. Subsequently, three exclusion phases were applied, resulting in the final selection of 560 articles included in the analysis. This methodical process ensured the relevance and quality of the literature examined.

## 3 Results

The results section presents the bibliometric analysis, wherein the principal trends and findings derived from the collated data are elucidated. This study will address key aspects such as the thematic evolution of the research, the identification of the most influential authors and journals, and the cooccurrence of keywords.

**Figure 5:**
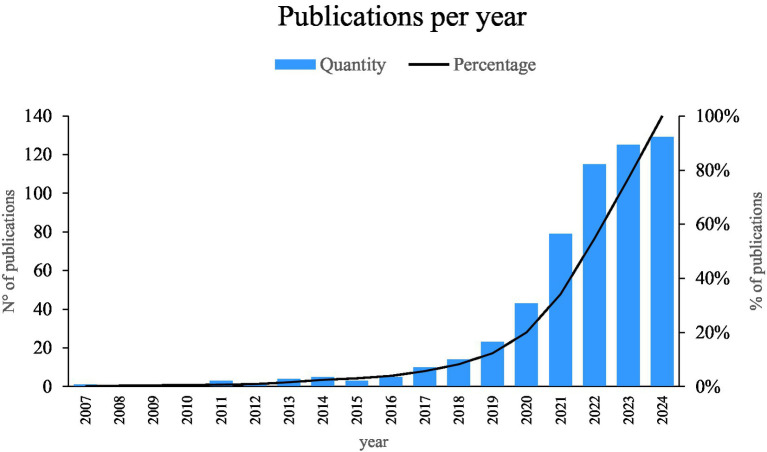
Publications per year. Prepared by the authors based on Scopus and Web of Science. Annual distribution of scientific publications related to the application of artificial intelligence in bacteriology from 2007 to 2024. Data were extracted from Scopus and Web of Science and visualized using Microsoft Excel®. The graph shows a clear upward trend, with exponential growth observed particularly from 2022 onwards. This trend highlights the increasing academic interest and research activity at the intersection of AI and microbiology.

As illustrated in Figure 5, the present bibliometry has enabled us to identify an exponential growth in scientific production on the use of artificial intelligence in bacteriology. This increase is particularly notable in the years 2024, 2023 and 2022, which suggests a growing interest and development in this intersection of knowledge. This increase in the number of publications may be indicative of both the advancement of artificial intelligence techniques and the necessity to more effectively address the challenges presented by contemporary bacteriology.

This increase in the number of publications may suggest a growing integration and practical maturation of artificial intelligence techniques within the field of bacteriology. While this study does not directly assess innovations in AI methodologies themselves, the upward trend in publications reflects that existing AI tools such as machine learning and deep learning are being increasingly adopted, adapted, and validated within bacteriological research. This suggests not only a wider accessibility of AI technologies but also a higher level of confidence in their applicability to domain-specific challenges. Furthermore, it points to stronger interdisciplinary collaboration between computer scientists and microbiologists, enabling AI methods to move beyond theoretical development and into practical, problem-solving contexts. Therefore, rather than interpreting this growth as an indicator of advancement in AI as a discipline, we frame it as evidence of the expanding utility, acceptance, and operational readiness of AI techniques within applied microbiology and bacteriology.

In the analysis of the principal authors, two distinct groups were identified. The first group comprises authors such as Singh A, Waegeman W and Vandamme P, who are regarded as authoritative figures despite exhibiting a relatively low level of scientific productivity. The second group comprises authors such as Wang Y and Kim S, who are distinguished primarily for their high scientific productivity, although their number of citations is relatively low.

**Figure 6:**
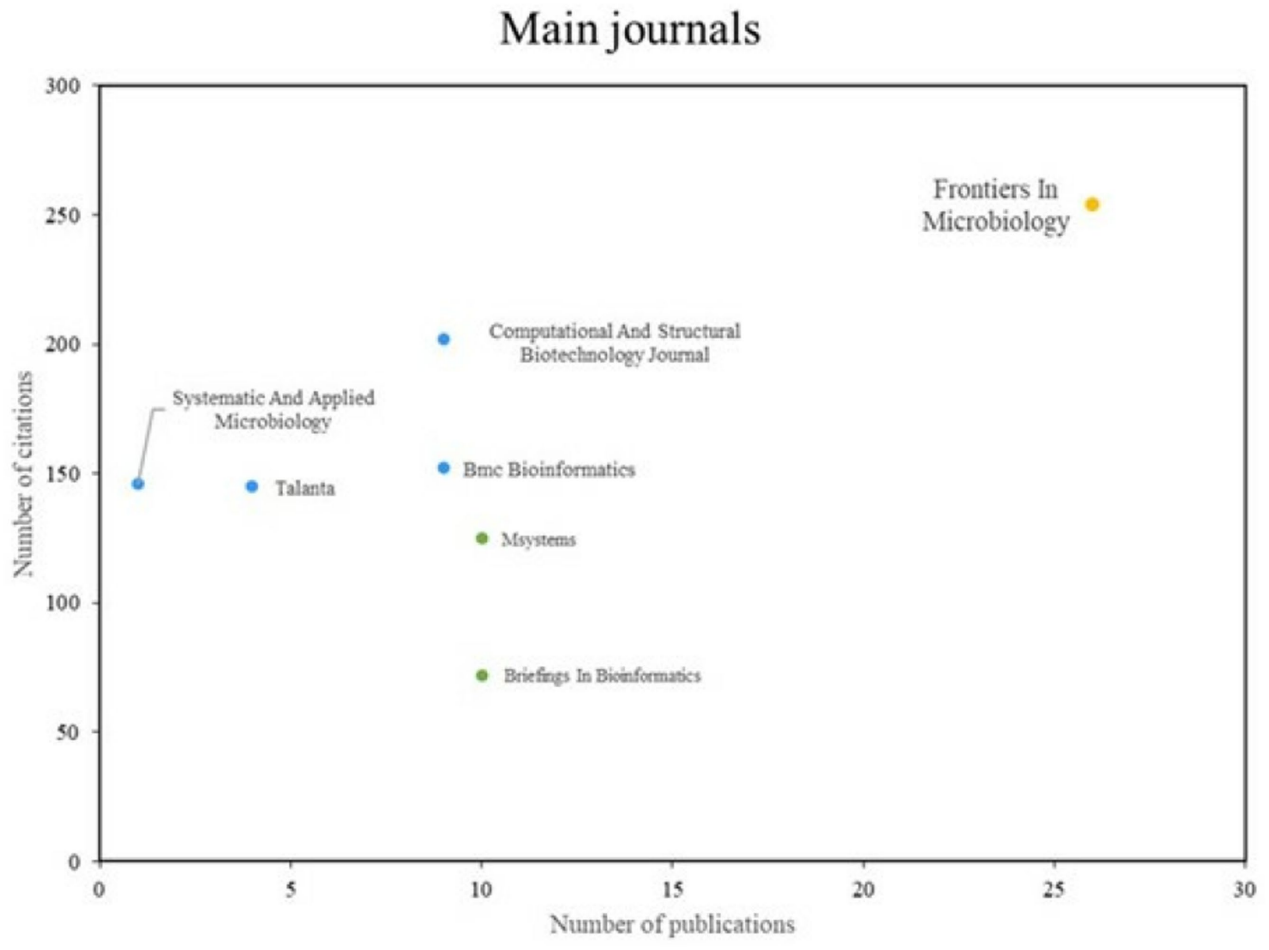
Main journals. Prepared by the authors based on Scopus and Web of Science. Distribution of the most relevant journals in terms of number of publications (X-axis) and citations (Y-axis) related to AI applications in bacteriology. The journals are grouped by performance and visualized through color coding: yellow for high productivity and high impact (e.g., *Frontiers in Microbiology*), blue for high impact but low productivity (e.g., *Computational and Structural Biotechnology Journal*), and green for high productivity but moderate impact (e.g., *MSystems*). This classification supports the identification of key publishing platforms in the domain.

In order to identify the most prominent journals, three distinct groups were identified, as illustrated in Figure 6. The initial group comprises journals that exhibit both high productivity and impact, with Frontiers in Microbiology serving as a notable exemplar. The second group is distinguished by high impact despite a relatively low index of scientific productivity, with the journal Computational and Structural Biotechnology Journal representing a notable example. The third group consists of journals that are recognized mainly for their scientific productivity, but not for the number of citations; MSystems is a particularly prominent example of this group.

To enhance interpretability, colors were used in Figure 6 to visually differentiate these groups:

*Yellow*: represents journals with both high productivity and impact (e.g., Frontiers in Microbiology).
*Blue*: indicates journals with high citation impact but lower publication output (e.g., Computational and Structural Biotechnology Journal, Systematic and Applied Microbiology).
*Green*: denotes journals with a relatively high number of publications but lower citation rates (e.g., MSystems, Briefings in Bioinformatics).

This color scheme supports a clearer understanding of how different journals contribute to both the volume and influence of AI-related research in bacteriology.

**Figure 7:**
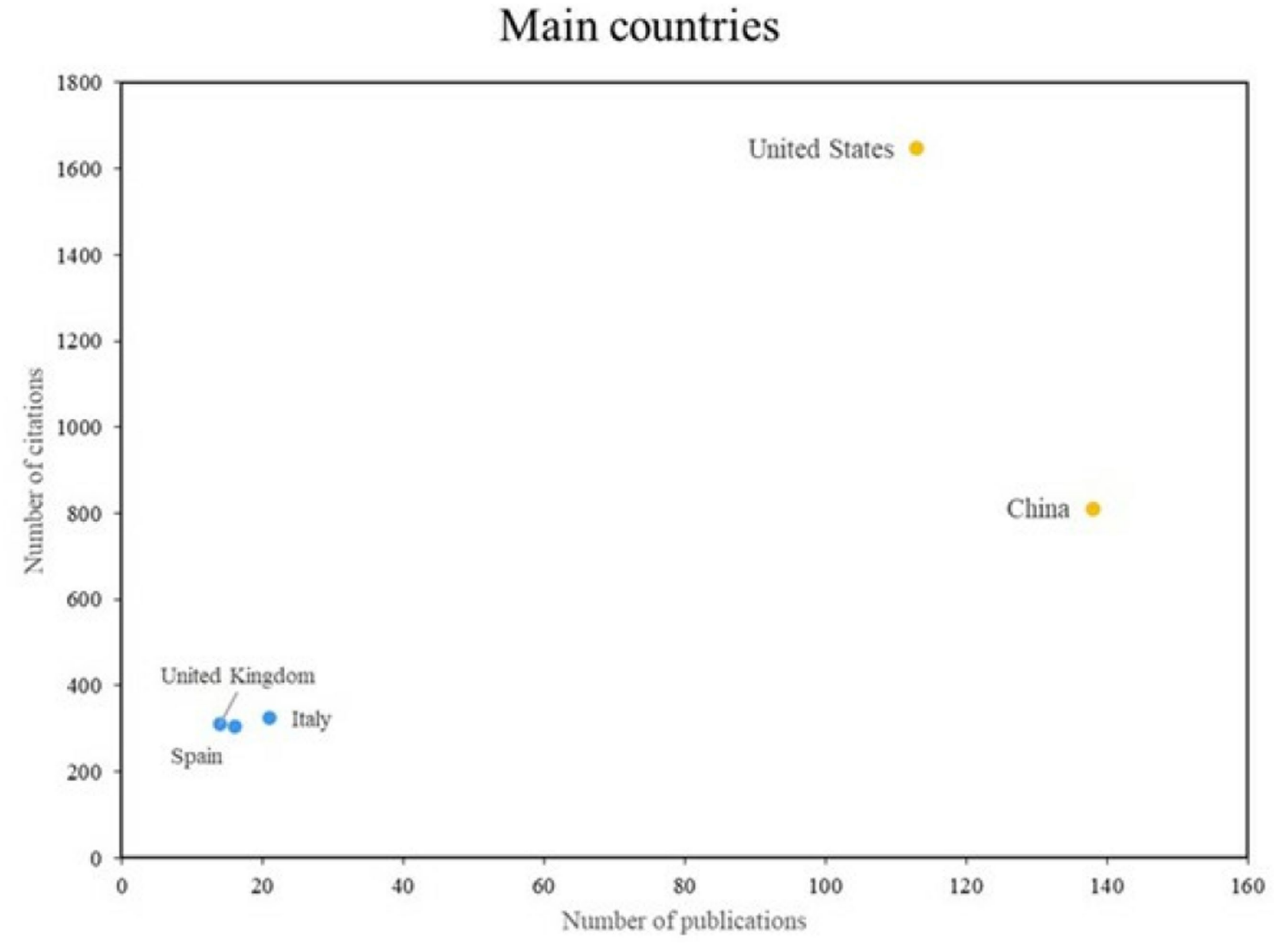
Main countries. Own elaboration based on Scopus and Web of Science. Countries with the highest scientific output and impact in the field of artificial intelligence applied to bacteriology. The data were retrieved from author affiliations in the Scopus and Web of Science databases. Two groups are observed: countries with both high productivity and impact (e.g., United States, China), and those with fewer publications but high citation rates (e.g., United Kingdom, Italy). The figure highlights geographical trends in research leadership and collaboration.

The analysis of the top countries revealed the existence of two distinct groups, as illustrated in Figure 7. The initial group comprises countries that exhibit a combination of high productivity and impact, with the United States and China representing notable examples. Conversely, the second group comprises countries such as the United Kingdom and Italy, which are regarded as exemplars in terms of impact despite exhibiting a relatively low index of scientific productivity.

As illustrated in Figure 3, the analysis of keyword frequency by year allows us to observe which topics were most prominent in each specific period. While the figure does not present a continuous or linear timeline, it provides insight into the conceptual focus of the field at different moments. For instance, earlier years (e.g., 2007) are marked by frequent terms such as “microbial spoilage,” while more recent years show a higher prevalence of terms like “bacteria classification,” “stage diagnosis,” and “microbiome.” This shift in dominant keywords across years reflects a broader thematic evolution and helps identify emerging research priorities in the application of AI to bacteriology.

**Figure 8:**
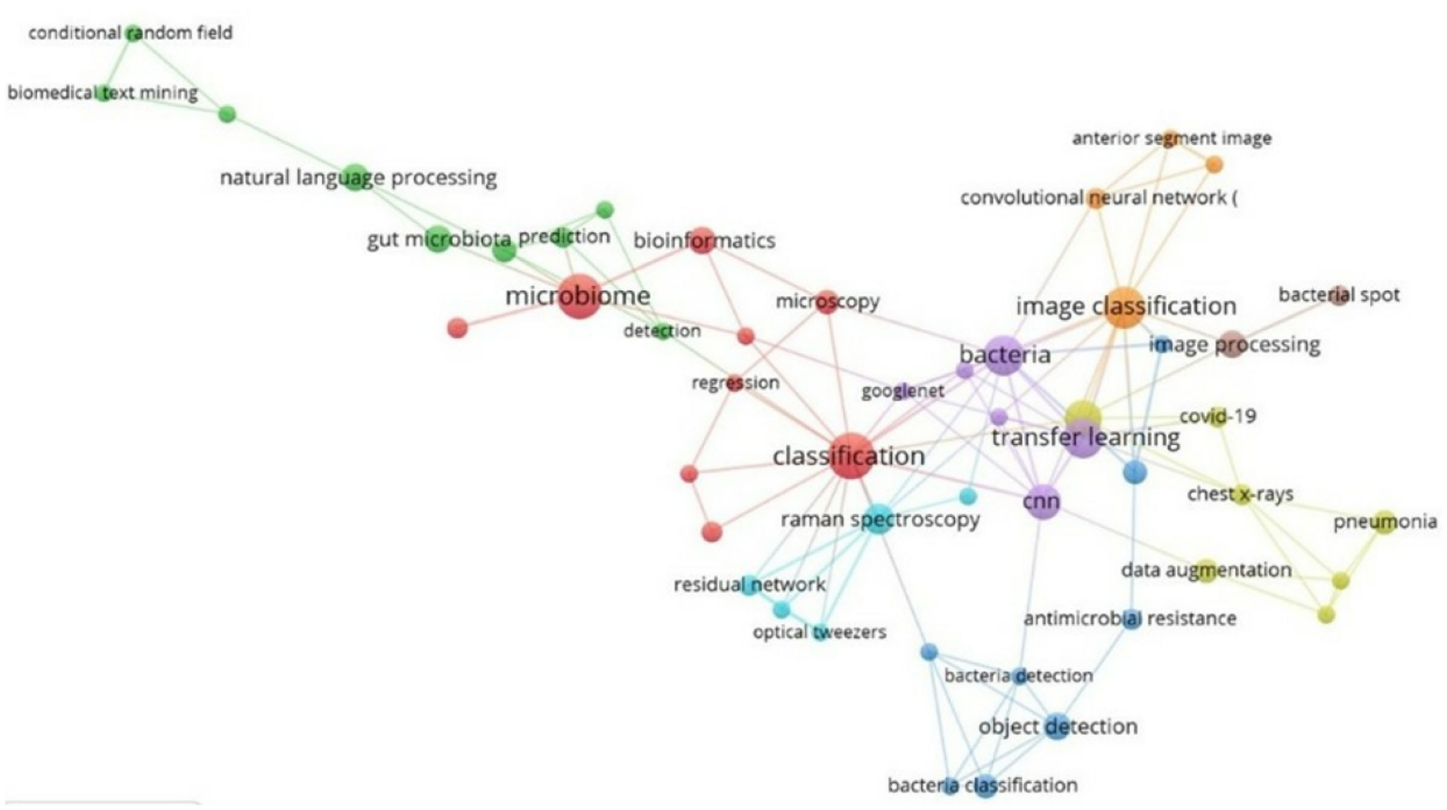
Keyword cooccurrence network. Own elaboration based on Scopus and Web of Science. Network visualization of keyword co-occurrence clusters in the analyzed literature, generated using VOSviewer. Each color represents a thematic cluster based on the co-occurrence frequency of terms. The red cluster (e.g., “classification,” “microbiome”) represents the core of current research, while others such as the purple cluster highlight emerging technical approaches like transfer learning and CNNs. This map reveals the conceptual structure and research directions in the field.

The principal keyword cooccurrence network is presented, comprising a total of eight thematic clusters, as illustrated in Figure 8. The red cluster, which includes terms such as “Classification,” “Microbiome,” “Bioinformatics” and “Microscopy,” is the most relevant, followed by the purple cluster, which covers keywords such as “Bacteria,” “Googlenet,” “Transfer Learning” and “CNN.” Furthermore, other clusters in blue, yellow, green and orange are identified, which reflect different elements of conceptual affinity in research on the use of AI in bacteriology.

This research proposes a Cartesian plane that measures the frequency of use of keywords on the Xaxis and the validity of use on the Y axis. This allows the visualization of four different quadrants, as seen in Figure 4. validity refers to the temporal relevance of a keyword, assessed through its average publication year, which indicates how recently the term has been used in the literature. In this scheme, the fourth quadrant is designated for concepts that are decreasing in frequency and current use within the literature. The graphic representation facilitates the identification of trends in research on the utilization of AI in bacteriology.

Keywords in quadrant 2 are highlighted as emerging concepts despite their infrequent occurrence and high validity. Such terms include “diagnosis,” “metagenomics,” “random forest,” and “transfer learning.” in quadrant 1 concepts that are both well established and experiencing growth are situated. an example of this is the term “microbiome,” which is both frequently used and remains a relevant topic in contemporary research. This classification enables the identification of areas of emerging and consolidated interest within the field

## 4 Discussion

In the discussion section, the results obtained in the research are subjected to a thorough analysis, while their practical implications are presented. Furthermore, the study’s limitations are discussed, and a research agenda is proposed, outlining the priority areas for future studies in this field.

### 4.1 Analysis of the growth of scientific literature on the use of AI in bacteriology

The analysis of the growth of scientific literature over time reveals a sustained upward trend in publications focused on the application of artificial intelligence in bacteriology. This trend becomes particularly notable from 2022 onward, coinciding with a broader global interest in AI-driven solutions across fields such as healthcare, biotechnology, and environmental monitoring. Rather than reflecting isolated contributions, the increase suggests a growing academic and institutional commitment to applying AI tools to solve bacteriological challenges. It also reflects enhanced access to data and computational resources, which have facilitated the operational use of machine learning and deep learning in microbiological contexts.

The thematic evolution of recent studies indicates a transition from exploratory analyses to more specialized and application-oriented research. In 2022, for example, a research team employed machine learning to investigate bacterial population dynamics and environmental diversity, providing insights into microbial responses under varying ecological conditions (Aida et al., 2022). In 2023, a machine learning-assisted electrochemical sensor was developed for the detection of three bacterial species, showcasing practical diagnostic potential (Wang et al., 2023). By 2024, the trend continued with the simulation of microbial communities for anaerobic digestion optimization using AI-based models (Yu et al., 2024). These examples illustrate how AI techniques are progressively moving from theoretical exploration to real-world application, signaling a phase of technological maturity and interdisciplinary convergence in the field.

### 4.2 Analysis of research references on the use of AI in bacteriology

The bibliometric analysis revealed several researchers whose contributions have significantly influenced the development of artificial intelligence applications in bacteriology. Among them, Waegeman W. and Vandamme P. stand out for their work on bacterial species identification through computational approaches. In particular, their study using machine learning with MALDI-TOF mass spectrometry data demonstrated how algorithmic analysis of spectral profiles can improve taxonomic resolution and reduce misclassification errors (Bruyne et al., 2011). This work provided a practical framework for integrating AI into microbiological diagnostics, especially where traditional methods reach their limitations.

Another notable contributor is Singh A., whose research focuses on deep learning for plant disease detection. In his study on peach leaf bacteriosis, Singh applied convolutional neural networks (CNNs) to image data to distinguish between infected and healthy leaves. While the study is in an agricultural context, it shows how deep learning can be adapted for the detection of bacterial pathologies based on subtle visual features, a method that holds potential for applications in clinical and environmental bacteriology as well (Yadav et al., 2021).

Wang Y. has contributed to the understanding of bacteriophage–host interactions using deep learning. His models predict phage–bacteria relationships from genomic sequences, which is critical for microbiome research and therapeutic strategies such as phage therapy. The methodological strength of his work lies in combining biological sequence analysis with deep learning classification, allowing accurate prediction of complex biological interactions (Li et al., 2020).

Kim S., in turn, has developed an innovative approach by integrating supervised machine learning with portable paper-based microfluidic devices. His work enables on-site bacterial species classification using smartphones, thus broadening access to diagnostic technologies. The main contribution of this research lies in the democratization of AI-driven diagnostics, enabling low-resource environments to perform microbial classification with minimal equipment and training (Kim et al., 2021).

Overall, these authors exemplify the diversity of AI applications within bacteriological research. Their work spans multiple subfields including taxonomic classification, image-based diagnostics, microbial ecology, and mobile health demonstrating how AI techniques can be tailored to address specific bacteriological challenges. More importantly, their studies highlight how interdisciplinary approaches integrating microbiology, computer science, and bioengineering are shaping the future of diagnostic innovation and microbial analysis.

Several of the leading countries identified in Figure 7 have implemented artificial intelligence (AI) in bacteriology through concrete and impactful applications. In the United States, for example, Thrift et al. (2020) used deep learning on vibrational spectral data to enable rapid antimicrobial susceptibility testing, which significantly accelerated diagnostic workflows. In China, Wang et al. (2023) developed a machine learning–assisted electrochemical sensor for detecting multiple bacterial species, enabling accurate, portable diagnostics.

The United Kingdom contributed to this field through the work of Bruyne et al. (2011), who used support vector machines to analyze MALDI-TOF mass spectra and improve bacterial species identification. In Italy, Ferrari et al. (2015) implemented convolutional neural networks (CNNs) to automatically count and classify bacterial colonies, thereby streamlining laboratory processes. India has also advanced AI applications in bacteriology. Yadav et al. (2021) used CNNs to detect bacterial leaf spot in peaches, demonstrating an agricultural application with potential translational value for clinical and environmental microbiology. These examples illustrate the diversity of approaches and reinforce the global nature of AI integration in bacteriological research.

All five countries have made significant contributions to the application of AI in bacteriology. However, there are notable differences in their research priorities, technological adoption, and scientific output. The United States and China are the leaders in the volume and impact of their publications. This is driven by their substantial investment in biomedical AI and their access to large-scale datasets. They tend to prioritize clinical diagnostics and high-throughput screening platforms. The United Kingdom emphasizes methodological rigor and algorithm development, particularly in spectral analysis and taxonomic precision. Italy’s contributions often relate to laboratory automation and computer vision, reflecting its strengths in medical imaging and engineering integration. India focuses on cross-disciplinary applications, particularly in agriculture and public health. The country leverages AI to address region-specific challenges with limited-resource solutions. These differences reflect disparities in funding and infrastructure as well as distinct national strategies, health priorities, and levels of interdisciplinary collaboration.

### 4.3 Analysis of the thematic evolution on the use of AI in bacteriology

In the nascent stages of AI research in bacteriology, the concept of “microbial spoilage” was a pivotal focus, with the objective of expediting the detection of microbial contamination in food products. This enabled the creation of pioneering techniques, such as FTIR spectroscopy in conjunction with machine learning, for the identification and quantification of harmful microorganisms in real time (Ellis et al., 2007).

At the present time, the classification of bacteria represents a pivotal subject of investigation. In the year 2024, a bacterial classification model utilizing the Big Transfer (BiT) technique has emerged as a notable advancement, demonstrating enhanced accuracy and efficiency in the identification of bacterial species (Visitsattaponge et al., 2024). In 2023, the concept of “stage diagnosis” emerged as a significant area of interest, with the development of a deep learning based approach to diagnose the progress of bacterial spot in soybeans (Wang et al., 2023).

### 4.4 Analysis of thematic clusters on the use of AI in bacteriology

In the analysis of the keyword co-occurrence network, thematic clusters were identified that reveal conceptual affinities as well as application trajectories. The primary cluster, indicated in red, encompasses terms such as classification, microbiome, bioinformatics, and microscopy. This cluster exhibits a strong alignment with the emerging scientific trend toward precision medicine and microbiome research, where machine learning has demonstrated potential to facilitate immune profiling of patients, thereby deepening the understanding of microbial colonization (Wang et al., 2021). Real-world applications include the integration of AI in microbiome-based diagnostics and personalized treatment strategies, which are particularly relevant for addressing antimicrobial resistance. At the policy level, this cluster underscores the necessity for regulatory frameworks to ensure the ethical management of patient microbiome data and the promotion of interoperable bioinformatics infrastructures (Feng et al., 2023).

The second cluster, indicated in purple, encompasses terms such as “bacteria,” “GoogLeNet,” “transfer learning,” and “CNN.” This cluster is indicative of the rapid expansion of AI-driven imaging analysis in bacteriology and clinical diagnostics. Advancements in the field, such as automated classification of chest X-rays for severe acute respiratory syndrome coronavirus 2 (SARS-CoV-2) and bacterial pneumonias (Tiwari et al., 2022), underscore the potential of deep learning models to expedite diagnostic workflows while preserving diagnostic precision. Furthermore, the continuous advancement of bacterial image classification systems (Abougarair et al., 2024) signifies a nascent trend in digital microbiology, wherein artificial intelligence has the potential to supplement or even supplant conventional culture-based methodologies. From a policy perspective, the diffusion of such technologies underscores the importance of investing in digital health infrastructure and workforce training, while also addressing issues of algorithmic transparency and equitable access to AI-driven diagnostics.

Taken together, these thematic clusters not only map the scientific evolution of AI applications in bacteriology but also indicate converging priorities for research funding and health policy. The red cluster emphasizes foundational research and translational opportunities in microbiome and immunological profiling, while the purple cluster highlights the clinical applicability of AI for bacterial detection and disease monitoring. Both clusters converge on the broader need for coordinated policy strategies that foster responsible innovation, ensure data protection, and bridge the gap between laboratory advances and healthcare implementation.

### 4.5 Analysis of the frequency and conceptual validity around the use of AI in bacteriology

In the analysis of the Cartesian plane, in quadrant 2, emerging concepts are identified in research on the use of AI in bacteriology, with particular emphasis on the areas of “diagnosis” and “metagenomics.” The integration of artificial intelligence in the field of diagnosis, particularly in relation to the intestinal microbiome, has demonstrated considerable promise. For instance, a machine learning strategy has been devised to diagnose cardiovascular diseases by analyzing the intestinal microbiome (Aryal et al., 2020).

The term “metagenomics” reflects the significant advancements in our comprehension of microbial composition and its implications for human health. Recent research has employed machine learning to analyze the intestinal microbiome in infants, identifying microbial species that are influential in the development of diseases (Fernández-Edreira et al., 2021).

In quadrant 1, consolidated concepts were identified, with “microbiome” being one of the most relevant. There has been significant progress in microbiome research, particularly in metagenomic studies that examine the relationship between microbial composition and agricultural productivity. A recent study demonstrates the potential of machine learning approaches in predicting crop productivity by associating with the soil microbiome (Chang et al., 2017).

In addition to thematic trends, the bibliometric results reveal the frequent appearance of specific artificial intelligence techniques that are increasingly being applied in bacteriology. Among these, machine learning stands out as the most widely adopted, particularly for classification tasks and predictive modeling. Techniques such as random forest, support vector machines (SVM), and convolutional neural networks (CNN) appear recurrently, especially in studies involving bacterial image analysis, diagnostic predictions, and metagenomic profiling. Over time, the presence of more advanced approaches like deep learning and transfer learning indicates a shift toward more sophisticated models capable of handling complex, high-dimensional microbiological data. These methods are frequently associated with emerging keywords such as “diagnosis” and “metagenomics,” illustrating how AI techniques support the resolution of real-world bacteriological challenges. This evolution not only reflects growing technical maturity but also validates the interdisciplinary integration of computer science into microbiological research practices.

The prevalence of traditional machine learning techniques, such as support vector machines (SVMs) and random forests (RFs), in the analyzed literature is due to their robustness, generalization capacity, and computational efficiency. These characteristics are especially important in bacteriological datasets with small sample sizes and high dimensionality (Bruyne et al., 2011). These techniques allow for precise classification with minimal preprocessing and are ideal for clinical and diagnostic applications requiring interpretability and reproducibility. Their dominant presence also reflects their maturity and consistent validation in microbiological applications over the past decade (Topçuoğlu et al., 2020).

In contrast, pretrained transformer-based foundational models, though emerging as state-of-the-art tools in genomic sequence analysis and metagenomics, are not yet prominently represented in the examined bibliometric records. This absence may be explained by their recent adoption, high computational demands, and frequent dissemination through preprints or conference proceedings, which were excluded from this study to maintain methodological rigor. Thus, the prevalence of SVM and RF does not indicate a lack of innovation but rather highlights the ongoing relevance and practical application of these models in the current bacteriological research landscape (Li et al., 2020).

Table 1 presents selected case studies that illustrate the practical relevance of AI in bacteriology by exemplifying specific technologies, their implementation, and their impact. These examples demonstrate the variety of AI applications and their advantages in terms of speed, accuracy, and accessibility.

**Table 1:** Examples of AI applications in bacteriology: developers, implementation, and significance.

Technology and implementation	Significance	Authors
Used SVM algorithms to classify bacterial species from MALDI-TOF mass spectrometry profiles. The model processed spectral data to enhance taxonomic resolution.	Increased diagnostic precision and reduced misclassification compared to conventional taxonomy. Enabled faster identification with minimal expert input.	Bruyne et al. (2011)
Developed a deep learning model to analyze vibrational spectra from bacterial lysates for rapid antimicrobial susceptibility testing.	Achieved real-time prediction of bacterial resistance patterns, accelerating clinical decision-making and supporting early intervention.	Thrift et al. (2020)
Designed a smartphone-based paper microfluidic system integrated with supervised machine learning to identify bacterial species on-site.	Provided low-cost, portable diagnostics suitable for low-resource settings. Enhanced accessibility and democratization of AI in bacteriology.	Kim et al. (2021)

Other recent studies have employed comparable artificial intelligence techniques to those analyzed in this work, which reinforces the relevance of these methods in bacteriological research. For example, Wu and Gadsden (2023) conducted a comparative evaluation of traditional machine learning algorithms, such as supporting vector machines and random forests, for microbial classification tasks. Their findings support the effectiveness of these models in structured biological datasets, which align with the current study’s observations about their continued usefulness in bacteriology. Shang et al. (2022) introduced a transformer-based model for identifying bacteriophages in metagenomic data. This model demonstrated improved accuracy and reduced false positives compared to conventional classifiers. Similarly, Ratcliff (2024) explored using transformer architectures to generate synthetic bacteriophage genomes. They found that AI-generated sequences were compositionally distinct from natural ones. This suggests a novel approach to studying genomic diversity and evolution. These studies support the growing interest in integrating transformer-based models in microbiological contexts. However, their representation in peer-reviewed, bacteriology-specific bibliometric literature remains limited.

Despite the methodological similarities in the use of AI techniques, this study differs from prior works in several key aspects. First, unlike Shang et al. (2022) and Ratcliff (2024), who focused on developing or evaluating transformer-based models for specific genomic applications, this study uses a bibliometric approach to map trends in scientific production, conceptual structures, and emerging research agendas in bacteriology. Second, this study’s scope is broader, encompassing multiple AI techniques, including traditional machine learning and deep learning, and their applications in bacterial classification, diagnostics, and metagenomics. Third, the temporal coverage spans from 2007 to 2024, enabling identification of long-term thematic shifts and growth patterns. Finally, this study focuses specifically on bacteriology as a subfield rather than general microbiology or genomic modeling, providing a targeted perspective on the integration of AI into bacterial research at a systemic level.

### 4.6 Theoretical implications

The analysis of publication frequency demonstrates a sustained growth in the utilization of AI techniques for the identification and classification of bacteria, indicating a maturation in research and a growing acceptance of AI in microbiology. The identification of theoretical referents, such as the authors Singh A and Waegeman W, and high impact journals such as Frontiers in Microbiology, serves to illustrate the interconnection between diverse lines of research. Thematic evolution demonstrates a shift in focus from “Microbial Spoilage” to contemporary topics such as “Bacteria Classification” and “Microbiome.” This reflects a change in research priorities, which seek to enhance understanding of microbial diversity and its interaction with health and the environment.

The keyword cooccurrence analysis reveals thematic clusters, including “Classification,” “Microbiome,” and “Metagenomics.” These clusters illustrate both thematic affinities and potential avenues for future research, as they highlight the integration of diverse aspects of microbial knowledge in the context of AI. Furthermore, there is a paucity of literature addressing areas such as ‘Stage Diagnosis’ and ‘Transfer Learning’. This highlights the necessity for greater interdisciplinary collaboration. An investigation of these deficiencies may facilitate a more comprehensive comprehension of bacteriology and its interconnection with artificial intelligence.

### 4.7 Practical implications

The application of artificial intelligence in bacteriology offers concrete benefits for clinical diagnostics. Techniques such as deep learning, support vector machines, and random forest are being used to improve the accuracy and speed of identifying bacterial strains, antibiotic resistance, and disease progression. These advances enable more effective patient treatment protocols and reduce the time needed for laboratory diagnostics, which is crucial in the management of infectious diseases.

In the educational domain, the increasing use of AI in microbiological research points to the need for updated training programs. Concepts such as “transfer learning” and “CNN” are becoming essential tools in bacteriological analysis, yet are still underrepresented in traditional microbiology curricula. Integrating data science, bioinformatics, and AI modeling into academic programs can better prepare students and professionals to navigate the evolving landscape of microbial research and diagnostics.

From an industrial perspective, AI techniques are enabling innovation in the development of microbiological products and technologies. In the food industry, for instance, AI-powered microbial detection systems contribute to improved quality control, spoilage prevention, and safety assurance. Similarly, in pharmaceuticals and biotechnology, AI helps optimize bacterial strain selection and process monitoring, leading to more efficient production pipelines and cost reductions.

Lastly, bibliometric insights can serve as strategic tools for decision-makers and technology developers. Identifying emerging techniques and thematic shifts allows stakeholders to anticipate future needs and invest in research and development accordingly. The findings from this study may guide public and private initiatives in promoting AI adoption in bacteriology, ensuring that innovation aligns with scientific trends and societal demands.

### 4.8 Limitations

While the present bibliometrics provide valuable foundations, they are not without limitations inherent to their methodological design. Firstly, the selection of databases, specifically Scopus and Web of Science, may have introduced a bias in the representation of the scientific literature. This is because other relevant databases might not have been considered, which could limit the exhaustiveness of the findings, as some significant publications in the area might be absent.

Similarly, the PRISMA-2020 methodology, although robust, is subject to the interpretation of the inclusion and exclusion criteria, which may result in variations in the quantity and quality of the selected studies. Furthermore, the utilization of software such as Microsoft Excel® and VOSviewer® for data analysis, while effective, may restrict the depth of qualitative analysis of the publications, as it primarily focuses on quantitative indicators. Additionally, the definitions of key terms and their categorization into growing and emerging words may be subjective and vary over time, potentially affecting the long term validity and relevance of the results.

A notable limitation of this study is the exclusion of non peer reviewed documents, such as preprints and conference proceedings, which are particularly prevalent in fields with high innovation, such as artificial intelligence. While this decision was intended to ensure the inclusion of high quality and validated research, it may have partially restricted the representation of emerging publications, thereby introducing a potential bias toward more established sources indexed in major databases.

This study has several methodological limitations that should be acknowledged. First, relying exclusively on Scopus and Web of Science as data sources may introduce selection bias, as other relevant databases (e.g., PubMed and Dimensions) were not considered. Second, the analysis was limited to metadata (titles, abstracts, and keywords), which excluded full-text content and may have omitted deeper methodological insights and reduced contextual richness. Third, while excluding preprints and conference proceedings ensures peer-reviewed quality, it may also lead to the omission of recent or cutting-edge studies, especially in rapidly evolving fields such as deep learning and transformer-based models. Finally, the study used a quantitative bibliometric approach. Although this approach is good for identifying trends and structural patterns, it does not capture qualitative aspects, such as theoretical depth, model validation rigor, or implementation challenges in applied contexts.

### 4.9 Future research directions

To overcome the limitations identified in this study, future research should take a more inclusive approach. This can be achieved by incorporating additional databases, such as PubMed, Embase, and Dimensions, to expand the scope of indexed publications. Including preprints and conference proceedings while applying rigorous quality filters may also help capture emerging methods, particularly in areas such as deep learning and transformer architectures. Additionally, integrating qualitative content analysis, such as full-text reviews or expert interviews, would improve the contextual interpretation of AI applications in bacteriology. Mixed methods designs combining bibliometric mapping with clinical case studies or experimental validations could provide a more comprehensive understanding of how AI techniques are implemented, evaluated, and translated into practice. These approaches would enrich methodological depth and bridge the gap between theoretical development and real-world application in bacterial diagnostics and microbial research.

### 4.10 Research agenda

In this context, a research agenda refers to a structured set of proposed topics that highlight knowledge gaps, emerging trends, and future research priorities. It serves as a strategic guide for researchers and institutions aiming to advance the field based on current scientific evidence. The concept of “bacteria” is central to the application of artificial intelligence in bacteriology, as it serves as the foundation for developing models aimed at identifying, classifying, and understanding bacterial species and their roles in health and disease. The term “bacteria” is of paramount importance in the utilization of AI in bacteriology, as it facilitates advancement in the identification and classification of bacterial species and in the comprehension of their role in health and disease. The creation of algorithms that integrate genomic and phenotypic data is anticipated to enhance the classification of microbial species and facilitate the investigation of interactions within complex microbial communities.

It is of great importance to conduct research on classification, as an accurate classification of bacterial species has significant implications for diagnostics and treatments. Artificial intelligence comprises areas such as machine learning, within which specific techniques such as neural networks, support vector machines, and decision trees have demonstrated efficacy in differentiating bacterial strains. Furthermore, approaches that integrate multi-omic data could be investigated to enhance classification capabilities in real time. These techniques allow researchers to uncover subtle genomic or phenotypic differences between strains that traditional methods may overlook. As a result, AI-based classification models can improve pathogen identification, guide targeted therapies, and support real-time monitoring in clinical and environmental contexts.

The term “Ulcerative Colitis” has gained prominence due to the established relationship between the intestinal microbiota and this disease. The application of AI has facilitated the identification of patterns in the composition of the microbiota of patients, which may ultimately inform the development of novel, personalized therapeutic strategies through the utilization of probiotics and dietary modifications.

The term “antibiotics” is of great importance in bacteriology, given the rise in antibiotic resistance. Artificial intelligence is employed to analyze resistance patterns and predict the efficacy of treatments. The development of predictive models that optimize treatment regimens is recommended.

Support vector machines (SVMs) constitute a robust supervised learning technique with demonstrated efficacy in bacteriological contexts. Their operation is based on the determination of an optimal hyperplane that maximizes the margin between data classes in high-dimensional feature spaces. This approach is particularly suitable for microbiological datasets characterized by limited sample size and high dimensionality, such as those derived from genomic, proteomic or spectroscopic analyses. In applications involving MALDI-TOF mass spectrometry, SVMs have reduced misclassification errors and improved taxonomic resolution in bacterial species identification.

Their integration with Raman spectroscopy has enabled rapid, label-free classification of bacterial strains, facilitating real-time diagnostic processes without the need for reagents or extensive preprocessing. The combination of mathematical rigor, generalization capacity and adaptability to various bacteriological data types of positions SVMs as a powerful methodological tool. Their relevance justifies their inclusion in future research agendas aimed at advancing bacterial classification systems and enhancing the precision of automated diagnostic frameworks.

Raman spectroscopy is a crucial technique for identifying microorganisms, offering reagent free molecular insights. The incorporation of AI into Raman spectral analysis enhances data interpretation, facilitating rapid and precise identification. It is recommended to promote studies that integrate this technique with machine learning models to address intricate microbial interactions and open new avenues of research in the diagnosis of bacterial infections.

## 5 Conclusion

A bibliometric analysis indicates a growing interest in the integration of AI technologies in bacteriological research, as evidenced by a significant increase in publications in 2023 and 2024. This exponential growth suggests that there has been an increase in investment and a shift in focus toward addressing complex problems in contemporary microbiology.

Notable research contributions include those of Singh A., Waegeman W., and Vandamme P. These studies have been published in the journals Frontiers in Microbiology and MSystems, which have emerged as key platforms for this field of research. The United States and China are emerging as dominant players in scientific production, which could foster international collaboration.

The evolution of the thematic focus has shifted from “Microbial Spoilage” to more intricate areas such as “Bacteria Classification” and “Stage Diagnosis,” reflecting a shift in research priorities and their application in public health. Thematic clusters were identified, including “Classification,” “Microbiome,” “Bioinformatics” and “Microscopy,” which demonstrate intersections in research and suggest a multidisciplinary approach.

In conclusion, the emergence of new keywords such as “Diagnosis,” “Metagenomics” and “Random Forest” emphasizes the necessity to prioritize these areas in the research agenda, thereby facilitating the advancement of knowledge and innovation in the utilization of AI in bacteriology.

## Data Availability

The datasets presented in this study are publicly available. This data can be found at: https://doi.org/10.5281/zenodo.14617537.
