# Supplementary material for: Bibliometric analysis of the application of artificial intelligence techniques in bacteriology: a PRISMA-guided research agenda
Source: Front Microbiol. 2025 Sep 16;16:1641967. doi: 10.3389/fmicb.2025.1641967 (PMC12481518; doi:10.3389/fmicb.2025.1641967)
Supplement: Supplementary file 1 [file Supplementary_file_1.docx]

Supplementary Material

# Supplementary Data

Supplementary Material should be uploaded separately on submission. Please include any supplementary data, figures and/or tables.

Supplementary material is not typeset so please ensure that all information is clearly presented, the appropriate caption is included in the file and not in the manuscript, and that the style conforms to the rest of the article.

# Supplementary Figures and Tables

## Supplementary Figures


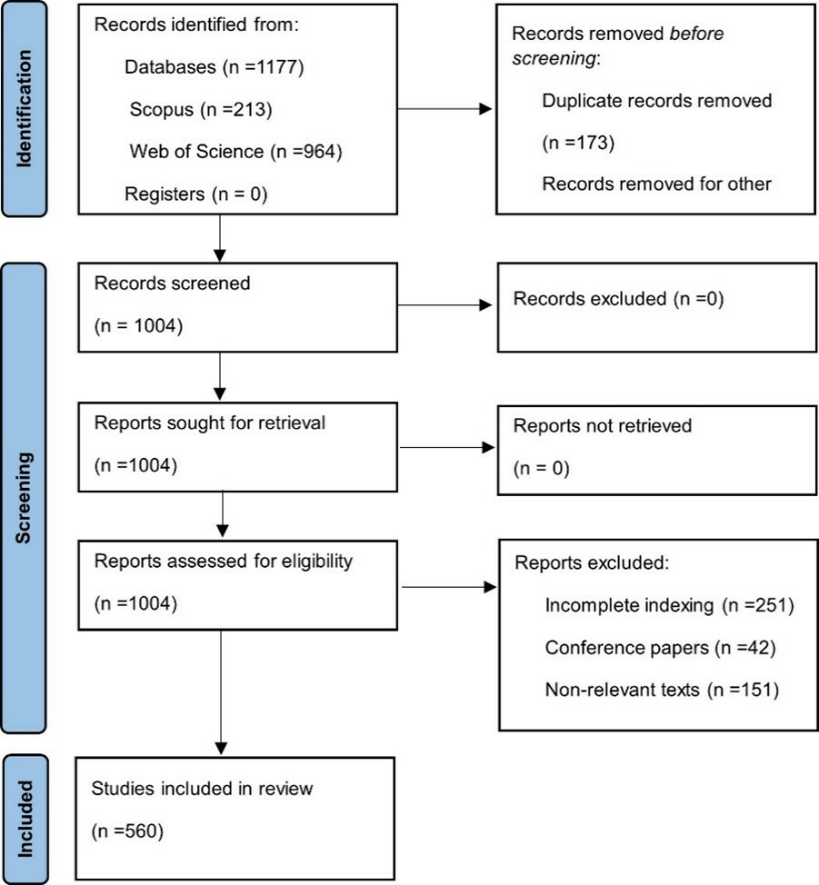
**Figure 1.** PRISMA flowchart. Own elaboration based on Scopus and Web of Science. PRISMA 2020 flow diagram showing the identification, screening, eligibility, and inclusion process of documents selected for the bibliometric analysis. Records were retrieved from Scopus and Web of Science using predefined search equations. The flowchart illustrates the systematic exclusion phases, including duplicate removal, metadata errors, and incomplete indexing. This ensures transparency in the selection process and methodological rigor in accordance with PRISMA guidelines.

**Figure 2**. Publications per year. Prepared by the authors based on Scopus and Web of Science. Annual distribution of scientific publications related to the application of artificial intelligence in bacteriology from 2007 to 2024. Data were extracted from Scopus and Web of Science and visualized using Microsoft Excel®. The graph shows a clear upward trend, with exponential growth observed particularly from 2022 onwards. This trend highlights the increasing academic interest and research activity at the intersection of AI and microbiology.

**Figure 3**. Main authors. Prepared by the authors based on Scopus and Web of Science. Most prolific and influential authors in the field of artificial intelligence applied to bacteriology, based on number of publications and citations. The data were processed from bibliographic metadata obtained through Scopus and Web of Science and analyzed using Excel®. The figure distinguishes between high-impact authors (with fewer but highly cited publications) and highly productive authors (with numerous publications but lower average citations), showing the different forms of scholarly influence in the field.


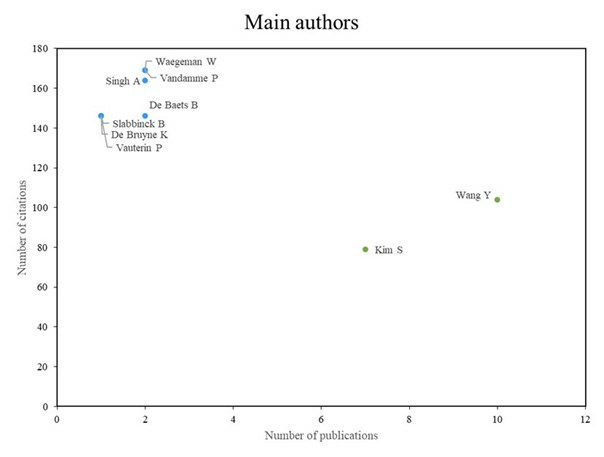


**Figure 4.** Main journals. Prepared by the authors based on Scopus and Web of Science. Distribution of the most relevant journals in terms of number of publications (X-axis) and citations (Y-axis) related to AI applications in bacteriology. The journals are grouped by performance and visualized through color coding: yellow for high productivity and high impact (e.g., *Frontiers in Microbiology*), blue for high impact but low productivity (e.g., *Computational and Structural Biotechnology Journal*), and green for high productivity but moderate impact (e.g., *MSystems*). This classification supports the identification of key publishing platforms in the domain.


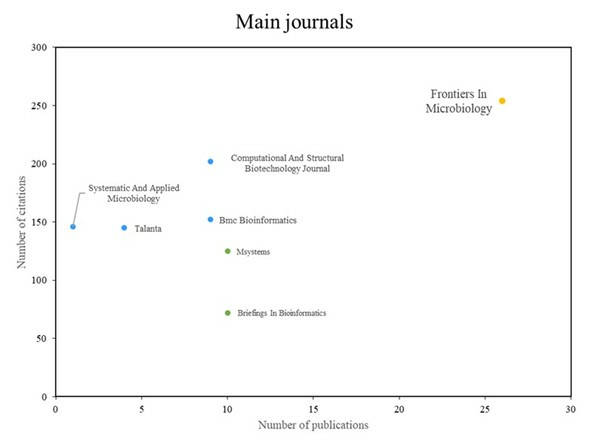


**Figure 5**. Main countries. Own elaboration based on Scopus and Web of Science. Countries with the highest scientific output and impact in the field of artificial intelligence applied to bacteriology. The data were retrieved from author affiliations in the Scopus and Web of Science databases. Two groups are observed: countries with both high productivity and impact (e.g., United States, China), and those with fewer publications but high citation rates (e.g., United Kingdom, Italy). The figure highlights geographical trends in research leadership and collaboration.


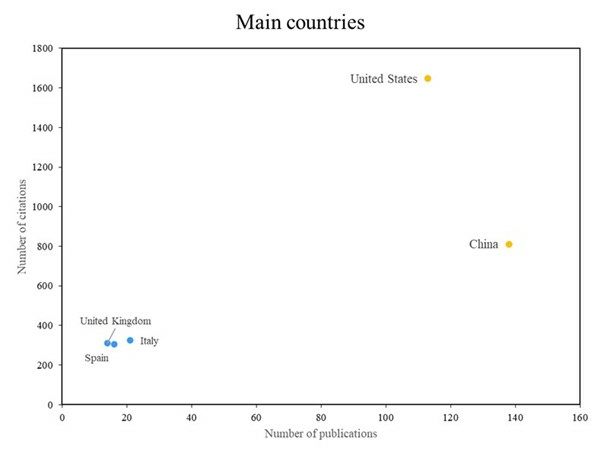


**Figure 6.** Thematic evolution. Own elaboration based on Scopus and Web of Science. Thematic evolution of keyword usage in publications related to AI in bacteriology from 2007 to 2022. Using VOSviewer, co-occurring terms were mapped over time to identify shifts in research focus. Early terms such as "microbial spoilage" have gradually been replaced by more advanced topics like "bacteria classification" and "microbiome." This figure illustrates the intellectual progression and growing complexity of the field.


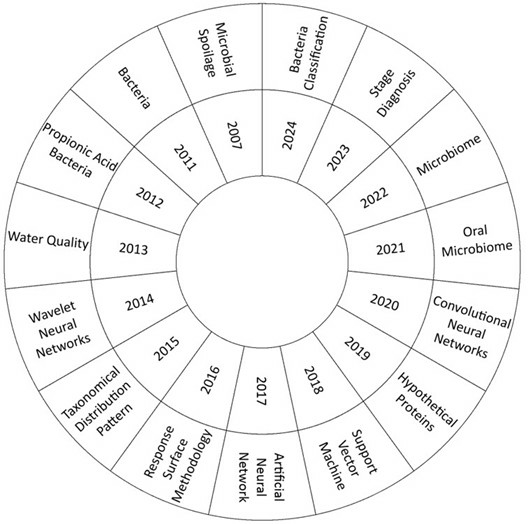


**Figure 7.** Keyword co-occurrence network. Own elaboration based on Scopus and Web of Science. Network visualization of keyword co-occurrence clusters in the analyzed literature, generated using VOSviewer. Each color represents a thematic cluster based on the co-occurrence frequency of terms. The red cluster (e.g., “classification,” “microbiome”) represents the core of current research, while others such as the purple cluster highlight emerging technical approaches like transfer learning and CNNs. This map reveals the conceptual structure and research directions in the field.


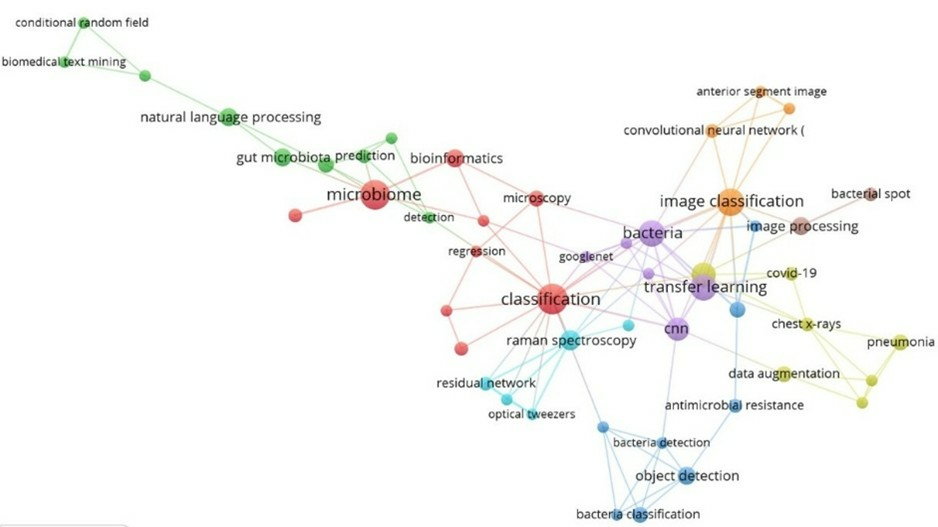


**Figure 8.** Validity and frequency of keywords. Prepared by the authors based on Scopus and Web of Science. Cartesian diagram mapping keywords according to frequency of use (X-axis) and temporal relevance (Y-axis). The figure classifies terms into four quadrants, with quadrant 1 showing established and growing topics (e.g., “microbiome”), and quadrant 2 highlighting emerging but underexplored concepts (e.g., “metagenomics,” “transfer learning”). This analysis helps identify research trends and gaps for future exploration


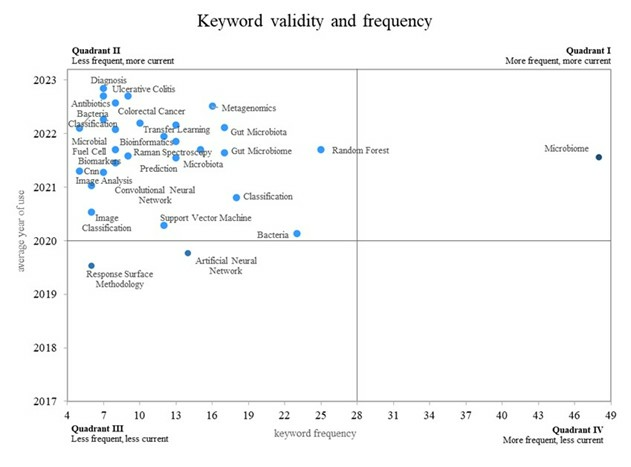


## Supplementary Tables

| **Technology and Implementation** | **Significance** | **Authors** |
| --- | --- | --- |
| Used SVM algorithms to classify bacterial species from MALDI-TOF mass spectrometry profiles. The model processed spectral data to enhance taxonomic resolution. | Increased diagnostic precision and reduced misclassification compared to conventional taxonomy. Enabled faster identification with minimal expert input. | Bruyne et al. (16) |
| Developed a deep learning model to analyze vibrational spectra from bacterial lysates for rapid antimicrobial susceptibility testing. | Achieved real-time prediction of bacterial resistance patterns, accelerating clinical decision-making and supporting early intervention. | Thrift et al. (13) |
| Designed a smartphone-based paper microfluidic system integrated with supervised machine learning to identify bacterial species on-site. | Provided low-cost, portable diagnostics suitable for low-resource settings. Enhanced accessibility and democratization of AI in bacteriology. | Kim et al. (28) |
